# 

**Published:** 1939

**Authors:** 


### The Medical Library of the University of Bristol

WITH WHICH IS INCORPORATED

**The Library of the Bristol Medico-Chirurgical Society.**

---

*The following donations have been received since the publication of the list in March,* 1939.

June, 1939.

American Laryngological Association (1) .. 2 volumes.
American Laryngological, Otological and Rhinological Society (2) .. .. .. 1 volume.
American Otological Society (3) .. .. 2 volumes.

| | |
|---|---|
| Professor E. W. Hey Groves (4) .. .. | 6 volumes. |
| London County Council (5) .. .. .. | 5 volumes. |
| London School of Hygiene and Tropical Medicine (6) .. .. .. .. | 1 volume. |
| Michell Clarke Fund (7) .. .. .. | 28 volumes. |
| Nitrate Corporation of Chile Ltd. (8) .. | 1 volume. |
| North Devon Athenæum (9) .. .. .. | 6 volumes. |
| H. Stuart Thompson, Esq. (10) .. .. | 1 volume. |
| Mr. E. Watson Williams (11) .. .. | 25 volumes. |

Unbound periodicals have also been received from Dr. R. F. Barbour, Professor E. W. Hey Groves, Dr. T. Milling, Professor C. Bruce Perry, Dr. G. de M. Rudolf, Dr. J. Odery Symes and Mr. E. Watson-Williams.

### THE ONE HUNDRED AND SIXTY-SEVENTH LIST OF BOOKS.

The figures in round brackets refer to the figures after the names of the donors. The books to which no such figures have been attached have either been bought from the Library Fund or received through the *Journal.*

| | | | |
|---|---|---|---|
| **Abernethy, J.** .. .. | *Physiological Lectures* .. .. .. .. | (9) | 1825 |
| **Bechterew, W. von**.. | *Die Leitungsbahnen im Gehirn und Rückenmark* .. .. .. | 2nd Ed. (11) | 1899 |
| **Beecher, H. K.** .. | *The Physiology of Anesthesia* .. .. .. | | 1938 |
| **Berkeley, Sir C., and others.** | *The Abnormal in Obstetrics* .. | (7) | 1938 |
| **Blackburn, A. E.** .. | *Cancer, Causation, Prevention and Treatment* | | 1939 |
| **Blech, G. M.** .. .. | *Clinical Electro-Surgery* .. .. .. .. .. | | 1938 |
| **Boyd, W.** .. .. .. | *Textbook of Pathology* .. .. | 3rd Ed. (7) | 1938 |
| **Bramwell, B.** .. .. | *Diseases of the Spinal Cord* .. .. | (11) | 1882 |
| **Brend, W. A.** .. .. | *Traumatic Mental Disorders in Courts of Law* .. .. .. .. .. .. .. .. .. | (7) | 1938 |
| **Brown, O. R.** .. .. | *Asthma* .. .. .. .. .. .. .. .. | (11) | 1917 |
| **Brumpt, E.** .. .. | *Précis de Parasitologie* .. .. | 4th Ed. (6) | 1929 |
| **Byrne, J. Grandson** .. | *Studies on the Physiology of the Eye*.. .. .. | | 1938 |
| **Cabot, R. C., and Adams, F. D.** | *Physical Diagnosis* | 12th Ed. (7) | 1939 |
| **Cameron, S. J. (ed.)** | *A Glasgow Manual of Obstetrics* | 3rd Ed. (7) | 1939 |
| **Carrel, A., and Lindbergh, C. A.** | *The Culture of Organs* .. .. | (7) | 1939 |
| *A Collection of all the papers relative to the alleged Adulteration of the Cheltenham Waters* .. .. .. .. .. .. .. .. .. .. .. .. | | | 1820 |
| **Collis, W. R. F. (ed.)** | *Clinical Pædiatrics.* (*The Baby*) .. | (7) | 1938 |
| **Conel, J. Leroy** .. | *The Postnatal Development of the Human Cerebral Cortex,* Vol. I. .. .. .. .. .. | | 1939 |
| **Cullen, W.** .. .. | *First Lines on the Practice of Physic,* 4 Vols. | New Ed. (9) | 1790 |
| **Davis, L.** .. .. .. | *Surgeon Extraordinary* .. .. .. .. .. | (7) | 1938 |
| **Dawkins, C. J. M.** .. | *On the Incidence of Anæsthetic Complications and their Relations to Basal Narcosis* .. | (11) | 1936 |

| | | |
|---|---|---|
| **Edridge-Green, F. W.** | *Memory, its Logical Relations and Cultivation* .. .. .. .. .. .. .. .. (11) | 1888 |
| **Findlay, G. M.** .. .. | *Recent advances in Chemotherapy* 2nd Ed. (7) | 1939 |
| **Fisher, A. G. T.** .. | *Treatment by Manipulation* 3rd Ed. (4) | 1939 |
| **Flack, M., and Hill, L.** | *A Text-book of Physiology* .. .. .. (11) | 1919 |
| **Flexner, S.** .. .. | *The Evolution and Organization of the University Clinic* .. .. .. .. .. .. .. .. | 1939 |
| **Garel, J.** .. .. .. | *Diagnostic et Traitement des Maladies du nez* .. .. .. .. .. .. .. 3rd Ed. (11) | 1910 |
| **Gib, C.** .. .. .. | *Vocal Science and Art* .. .. .. .. (11) | 1911 |
| **Glaister, J.** .. .. | *Medical Jurisprudence and Toxicology* 6th Ed. (7) | 1938 |
| **Gordinier, H. C.** .. | *The Gross and Minute Anatomy of the Central Nervous System* .. .. .. .. .. (11) | 1900 |
| **Hadfield, G., and Garrod, L. P.** | *Recent advances in Pathology* 3rd Ed. (7) | 1938 |
| **Hall, F. de Havilland.** | *Diseases of Nose and Throat* .. .. (11) | 1894 |
| **Handfield-Jones, R. M., and Porritt, A. E.** | *The Essentials of Modern Surgery* .. .. .. .. .. .. (7) | 1938 |
| **Harrison, G. A.** .. | *Chemical Methods in Clinical Medicine* 2nd Ed. (7) | 1937 |
| **Harrower, H. R.** .. | *Manual of Pluriglandular Therapy* .. (11) | 1924 |
| **Hellner, Hans** .. .. | *Unfall und Knochengeschwulst* .. .. (4) | 1939 |
| **Herman, L.** .. .. | *The Practice of Urology* .. .. .. (7) | 1938 |
| **Hill, Sir L., and Ellman, P.** | *The Rheumatic Diseases* .. .. (7) | 1938 |
| **Hosford, J.**.. .. .. | *Fractures and Dislocations in General Practice* | 1939 |
| **Hulbert, H. H.** .. | *Breathing for Voice Production* .. .. (11) | 1903 |
| **Jamieson, W. A.** .. | *Diseases of the Skin* .. .. 2nd Ed. (11) | 1889 |
| **Johnston, T. B.** .. | *Gray's Anatomy* .. .. .. 27th Ed. (7) | 1938 |
| **Knaus, H.** .. .. .. | *Periodic Fertility and Sterlity in Woman* .. | 1934 |
| **Lake, R.** .. .. .. | *International Directory of Laryngologists and Otologists*.. .. .. .. .. .. 2nd Ed. (11) | 1901 |
| **Langley, J. N.** .. .. | *Practical Histology* .. .. .. .. .. .. (11) | 1901 |
| **Leriche, R.** .. .. | *The Surgery of Pain* .. .. .. .. .. .. | 1939 |
| **Lilly, Eli., and Co.** .. | *The Anemias* .. .. .. .. .. .. .. .. (11) | 1938 |
| **Loeb, H. W.** .. .. | *Operative Surgery of the Nose, Throat and Ear.* 2 Vols. .. .. .. .. .. .. (11) | 1914 |
| **McClendon, J. F.** .. | *Iodine and the Incidence of Goiter* .. .. | 1939 |
| **MacLeod, A. C.** .. | *Some Radium Cases at the Middlesex Hospital* .. .. .. .. .. .. .. .. .. (11) | 1931 |
| **Magee, J. A. L.** .. | *Uterus Masculinus* .. .. .. .. .. .. .. | 1939 |
| **Martindale, W.** .. | *The Extra Pharmacopœia.* Vol. II. 21st Ed. (7) | 1938 |
| **Miller, S. C.** .. .. | *Text-book of Periodontia* .. .. .. (7) | 1938 |
| **Mitchiner, P. H., and others.** | *Surgery for Dental Students* .. (7) | 1936 |
| **Monrad-Krohn, G. H.** | *The Clinical Examination of the Nervous System* .. .. .. .. .. .. .. 7th Ed. | 1938 |
| **Moure, E. J.** .. .. | *Galerie Internationale des Oto-laryngologistes* (11) | 1908 |
| **Munro, D.** .. .. .. | *Cranio-Cerebral Injuries* .. .. .. .. .. .. | 1938 |
| **Packard, F. R.** .. | *Text-book of Diseases of the Nose, Throat and Ear* .. .. .. .. .. .. .. 2nd Ed. (11) | 1913 |

**Parker, C. A., and Faulder, T. J.** *Pharmacopœia of the Hospital for Diseases of the Nose, Throat and Ear* 7th Ed. (11) 1914

**Parrel, G. de** .. .. *Précis de Therapeutique Medicale Oto-rhino-laryngologique* .. .. .. .. .. .. (11) 1921

**Parsons, T. R.** .. .. *Fundamentals of Biochemistry* 5th Ed. (7) 1937

**Pemberton, R.** .. .. *Arthritis and Rheumatoid Conditions* .. (11) 1930

**Power, D'Arcy** .. .. *British Medical Societies* .. .. .. .. .. 1939

*Queen Charlotte's Text-book of Obstetrics* .. .. .. .. .. 5th Ed. (7) 1939

**Rogers, L., and d'Abreu, A. L.** *Everyday Surgery* .. .. .. .. (7) 1938

**Ryan, M.** .. .. .. .. *An Enquiry into the Nature, Cause and Cure of Consumption of the Lungs* .. .. (9) 1787

**Saeggesser, M.** .. .. *Das Panaritium* .. .. .. .. .. .. .. (4) 1938

**Sheldon, W.** .. .. *Diseases of Infancy and Childhood* 2nd Ed. (7) 1938

**Simpson, W.** .. .. *Hydrologia Chymica* .. .. .. .. .. .. .. 1669

**Thomas, J.** .. .. .. *Practical remarks on Chronic Affections of the Digestive Organs* .. .. .. .. 2nd Ed. 1821

**Thomson, St. Clair** .. *Diseases of the Nose and Throat* .. .. (11) 1911

**Thorek, M.** .. .. *Modern Surgical Technik.* 3 Vols. .. (4) 1938

**Thorpe, W. V.** .. .. *Biochemistry for Medical Students* .. (7) 1938

**Tidy, H. L.** .. .. *A Syopsis of Medicine* .. .. .. .. 7th Ed. 1939

*Treatment in General Practice.* Vol. III.*: Anæsthesia ; Surgery* .. 1939

**Walker, N., and Percival, G. H.** *Introduction to Dermatology* 10th Ed. 1939

**Wellings, A. W.** .. *Practical Microscopy of the Teeth and Associated Parts* .. .. .. .. .. .. (7) 1938

**White, C (ed.)** .. .. *Diseases of Women by Ten Teachers* 6th Ed. (7) 1938

**White, C. (ed.)** .. *Midwifery by Ten Teachers* 6th Ed. (7) 1938

**Willis, L. G.** .. .. *Bibliography of the Minor Elements* 3rd Ed. (8) 1939

**Wood, N. (ed.)** .. .. *Health Resorts of the British Islands* 2nd Ed. (10) 1919

### TRANSACTIONS, REPORTS, JOURNALS, ETC.

Collected Papers of the Mayo Clinic. Vol. XXX. .. .. (7) 1938

London County Council :—Measles. Report of the Medical Officer of Health on the Measles Epidemics, 1927–28, 1929–30, 1931–32, 1933–34, 1935–36 .. .. .. .. .. .. .. .. .. .. .. (5) 1929–38

Medical Annual, 1939 .. .. .. .. .. .. .. .. .. .. .. .. .. 1939

Modern Treatment Year Book, 1939 .. .. .. .. .. .. .. .. .. 1939

Quarterly Cumulative Index Medicus. Vol. 24. July–December, 1938 1939

Transactions of the American Laryngological Association. Vols. 59 and 60 .. .. .. .. .. .. .. .. .. .. .. .. .. .. .. (1) 1937–39

Transactions of the 48th Annual Meeting of the American Laryngological, Rhinological and Otological Society .. .. (2) 1938

Transactions of the American Otological Society. Vols. XXVII–XXVIII .. .. .. .. .. .. .. .. .. .. .. .. .. .. (3) 1938

### Publications Received

From Messrs. Edward Arnold & Co.:

*Physiotherapy in Medical Practice.* By Hugh Morris, M.D., D.M.R.E. Price 12s. 6d.

From Messrs. Cassell & Co. Ltd.:

*Surgical Applied Anatomy.* By Sir Frederick Treves, Bart. 10th Edition. Revised by Lambert Rogers, M.Sc., F.R.C.S., F.R.C.S.E., F.R.A.C.S., F.A.C.S. Price 14s.

From Messrs. W. Green & Son Ltd.:

*An Introduction to Dermatology.* By Norman Walker, Kt., M.D., LL.D. F.R.C.P., and G. H. Percival, M.D., Ph.D., F.R.C.P. 10th Edition. Price 20s.

From Messrs. H. K. Lewis & Co. Ltd.:

*Diathermy, Short Wave, Inductothermy, Epithermy,* By William Beaumont, M.R.C.S., L.R.C.P. Price 10s. 6d.

*Cancer: Causation, Prevention and Treatment.* By A. E. Blackburn, M.D. Price 6s.

*The Streptococcal Tendency.* By J. D. Hindley-Smith, M.A., B.Ch., M.R.C.S., L.R.C.P. Price 1s.

*Catalogue of Lewis's Medical and Scientific Lending Library.* Part II. Classified index of subjects with names of authors who have written upon them. Price complete 16s.

*Aequanimitas, with other addresses to Medical Students, Nurses and Practitioners of Medicine.* By Sir William Osler, Bart., M.D., F.R.S. Reprinted from the 3rd Edition. Price 7s. 6d.

*The Infant—a Handbook of Management.* By Wilfred J. Pearson, D.S.O., M.C., D.M., F.R.C.P., and Arthur G. Watkins, M.D., M.R.C.P., B.Sc. 2nd Edition. Price 2s. 6d.

*Royal Northern Operative Surgery.* By the Surgical Staff of the Royal Northern Hospital. Price £2 2s.

From Oxford University Press:

*Pulmonary Tuberculosis.* By George Gregory Kayne, M.D., M.R.C.P., D.P.H., Walter Pagel, M.D., and Laurence O'Shaughnessy, M.D., F.R.C.S. Price £2 2s.

*Iodine and the Incidence of Goiter.* By J. F. McClendon. Price £2 2s. 6d.

*The Heart Sounds in normal and Pathological Conditions.* By Oscar Orías, M.D., and Eduardo Braun-Menéndez, M.D. Price 15s.

*Pulmonary Tuberculosis—a Synopsis.* By James Segal, M.D., Price 10s. 6d.

From Messrs. John Wright & Sons Ltd.:

*Epidemiology in Country Practice.* By William Norman Pickles, M.D. Price 7s. 6d.

*Genetics and the Clinician.* By Lindsay Ride, M.A., B.M., B.Ch., M.R.C.S., L.R.C.P. Price 10s. 6d.

### Local Medical Notes

The twenty-eighth Long Fox Memorial Lecture will be delivered at the University on Tuesday, November 14th, 1939, by Dr. Clement C. Chesterman. Subject "Forest Folk: Modern Medicine in the Congo."

APPOINTMENTS.

**Bristol Royal Infirmary and Bristol General Hospital** (Amalgamated).—Honorary Anæsthetists: H. B. Logan, M.R.C.S., L.R.C.P., D.A.; E. G. Bradbeer, M.R.C.S., L.R.C.P., D.A. Honorary Assistant Obstetrician: W. M. Capper, M.B., B.S. (Lond.), F.R.C.S. (Eng.), M.R.C.O.G.

**Bristol General Hospital.**—Honorary Consulting Surgeon: C. A. Moore, M.S. (Lond.), F.R.C.S. (Eng.); Honorary Consulting Anæsthetist: C. Corfield, M.D. (Durh.), D.A., Barrister-at-Law.

**Bristol Royal Infirmary.**—Honorary Consulting Anæsthetist: L. A. Moore, M.B., Ch.B. (Brist.).

EXAMINATION RESULTS.

**University of Bristol.**—Students of the University have recently passed the following examinations:—

M.D.—Constance I. Ham, E. Philipp (by dissertation).

M.B., Ch.B.—*First Examination. Pass:* J. A. Ardis, R. I. Bodman, A. M. Brown, Nina Dawson-Reid, Marjorie J. Dix, Margaret P. Drew, Julia Fraser, E. C. Hamlyn, Kathleen C. Iles, Flavia Z. L. B. James, D. M. M. Jones, M. H. Kerby, Josephine C. Mulcahy, M. J. Mulcahy, W. Oldham, D. V. Pleasant, C. T. Ross, R. J. Sandry, P. A. Titterton, A. S. Wallace.

M.B., Ch.B.—*Second Examination. Pass:* Nancy R. Baldwin, R. F. Brooks, Rosalie M. Child, D. G. Davies, J. Deighton, Mary D. Dixon, G. F. M. Hall, Elisabeth Hodson, A. G. H. Mitchinson, E. W. Moore, J. L. Pardoe, E. S. Short, N. Slade, M. H. Thomas, G. H. Valentine, Jean Wallace, G. H. Wattley.

M.B., Ch.B.—*Final Examination.* (*Section I.*) *Pass:* Marjorie Dainton, Joan M. Foss, J. E. Smith.

M.B., CH.B.—*Final Examination. Pass (with Second Class Honours):* Dorothy M. Shotton (2, 3), C. A. St. Hill (2). *Pass:* Dorothy M. Ayre, P. A. Bennett, A. A. K. Datta, Marjorie O. Dunster (1), J. L. Elliott, J. L. Emery, Emily G. Hamlyn, Betty F. Hannaford (1, 2), Monica L. Hawkins, F. R. Hurford, N. E. Melling, Jeannette D. Shed (2), S. J. Silvey, P. R. H. Slade, Sylvia E. Smith, T. H. White.

B.D.S.—*First Examination. Pass:* Jean A. M. C. Gordon-Ralph, R. D. Hepburn, Alice B. U. Leigh, R. L. Yendall.

B.D.S.—*Second Examination. Pass:* C. H. Gurd, F. G. Lloyd, F. M. Warren.

B.D.S.—*Third Examination. Pass:* F. C. Bryant, P. B. Carey, Margaret J. Griffith, G. K. G. Jennings (4, 5), Sarah Jones, P. M. Nicholas (4), R. M. Sharp.

B.D.S.—*Final Examination. Pass:* G. H. Bell (6), J. E. H. Bell, J. F. Blenkinsop, R. J. Gething, A. A. Grant (1), D. R. Stevens.

L.D.S.—*First Examination. Pass:* J. W. B. Kay, B. A. Oliver.

L.D.S.—*Second Examination. Pass:* G. E. Barnard, Patricia M. Burke, G. G. Davis, I. E. Perry.

L.D.S.—*Third Examination. Pass:* Jean M. Bryant, D. H. Buckland, R. L. Hollington, G. J. Mills, B. N. Neilson, R. S. Saxton, V. T. Scard.

L.D.S.—*Final Examination. Pass:* F. W. Clift, K. A. Gordon-Ralph, G. H. Hudson, E. L. Manton, R. H. G. Sims, Mary E. Thomas, May A. Trump, L. N. Weale, H. D. Williams, E. S. Wolfson.

(1) With Distinction in Surgery. (2) With Distinction in Public Health.
(3) With Distinction in Forensic Medicine and Toxicology.
(4) With Distinction in Dental Mechanics and Properties of Metals.
(5) With Distinction in Dental Anatomy.
(6) With Distinction in Dental Pathology and Bacteriology.

**Conjoint Board.**—L.R.C.P., M.R.C.S.—*First Examination* (Pharmacology and Materia Medica). *Pass:* Joan M. Foss, C. J. R. Jacob, J. H. G. Morris.

L.R.C.P., M.R.C.S.—*Final Examination. Pass:* A. L. T. Beddoe (8), Betty Fox (8, 9), F. R Hurford (9), Megan E. Jones (10), Joyce S. Miller (9, 10), Mary R. More (9), A. A. Stonehill (7, 9), E. W. J. Townsend (10).

L.D.S., R.C.S.— *Part I.:* S. G. Sheffield.

L.D.S., R.C.S.—*First Professional Examination. Pass:* R. M. Sharp (Dental Mechanics and Dental Metallurgy).

(7) Medicine. (8) Surgery. (9) Midwifery. (10) Pathology.

